# Chromium and nickel in *Pteridium aquilinum* from environments with various levels of these metals

**DOI:** 10.1007/s11356-014-3379-5

**Published:** 2014-08-05

**Authors:** Kamila Kubicka, Aleksandra Samecka-Cymerman, Krzysztof Kolon, Piotr Kosiba, Alexander J. Kempers

**Affiliations:** 1 Department of Ecology, Biogeochemistry and Environmental Protection, Wrocław University, ul. Kanonia 6/8, 50-328 Wroclaw, Poland; 2 Institute for Water and Wetland Research, Department of Environmental Science, Radboud University Nijmegen, Heyendaalseweg 135, 6525 AJ Nijmegen, The Netherlands

**Keywords:** Bioaccumulation, Rhizome, Frond, Metal, Granite, Serpentinite

## Abstract

*Pteridium aquilinum* is a ubiquitous species considered to be one of the plants most resistant to metals. This fern meets the demands for a good bioindicator to improve environmental control. Therefore, it was of interest to survey the accumulation of Cr and Ni in the rhizome and fronds of this species collected in Lower Silesia (SW Poland) of serpentinite rich in Cr and Ni and granite poor in these metals. Additionally, concentrations of Cd, Co, Cr, Cu, Fe, Mn, Ni, Pb, and Zn were measured in granite and serpentinite parent rocks, soils, and in *P. aquilinum* (rhizome and fronds). The experiment was carried out with rhizomes of ferns from both types of soils placed in pots supplemented with 50, 100, and 250 mg kg^−1^ of Cr or Ni or both elements together. At a concentration of 250 mg kg^−1^ of Cr, Ni, or Cr + Ni, fronds (from granite or serpentinite origin) contained significantly higher Cr and Ni concentrations when both metals were supplied together. In the same concentration of 250 mg kg^−1^ of Cr, Ni, or Cr + Ni, rhizomes (from granite or serpentinite origin) contained significantly higher Cr and Ni concentrations when both metals were supplied separately. The explanation of metal differences in the joint accumulation of Cr and Ni on the rhizome or frond level needs further investigation. The lack of difference in Cr and Ni concentration in the rhizome and fronds between experimental *P. aquilinum* collected from granite and serpentinite soils may probably indicate that the phenotypic plasticity of this species is very important in the adaptation to extreme environments.

## Introduction

Ferns have often been associated with contaminated soils, particularly with mining operations (Samecka-Cymerman et al. 2012). Nevertheless, these plants have received less attention than vascular plants in relation to metal tolerance and accumulation (Kachenko et al. 2007; Niazi et al. 2012). Some fern species are important bioindicators of metalliferous soils or have the exceptional capability to hyperaccumulate some metals and have been used to decontaminate polluted sites (Kachenko et al. 2007; Leung et al. 2007; Zhang et al. 2012). Among fern species, *Pteridium aquilinum* (L.) Kuhn (bracken) is one of the world’s most successful and widely dispersed species, the only terrestrial fern that dominates large tracts of land outside woodland in temperate climates (Marrs and Watt 2006; Chang et al. 2009). The *P. aquilinum* common in disturbed habitats is also recognized as one of the plants most resistant to metals (Chang et al. 2009). Therefore, this fern meets the demands for a good bioindicator which could be used to enable environmental control (Markert et al. 2003). The amount of any metal taken up by plants from contaminated soils has been suggested as being of central importance in assessing the risk of toxicity (Roy and Gunjan 2010). There is extensive information on the contamination of soils and plants by single metals; however, the combined pollution with these elements is a more common phenomenon in nature with mitigating or amplifying effects (Pivetz 2001; Haiyan 2003). Because xenobiotics like Cr and Ni are usually present together in polluted areas, a selection was made of model environments of granites (usually poor in both metals) and serpentinites typically with high concentrations of both Ni and Cr (Galardi et al. 2007; Samecka-Cymerman et al. 2009). Under experimental conditions, the single and combined accumulations of Cr and Ni in the fern were compared. It is well known that plants have a capacity to adapt to certain environmental conditions (Fernándèz et al. 2000). Therefore, *P. aquilinum* from granite areas should accumulate significantly less Cr and Ni than those from serpentinite sites which live on soils with an excess of these elements. The aim of this study was to experimentally compare the concentration of Cr and Ni in the rhizome and fronds of *P. aquilinum* from serpentinite and granite sites with supplementation of both elements separately and in combination. The following hypotheses were investigated: (1) combined concentrations of Cr and Ni supplied to *P. aquilinum* may cause suppressing effects on the sum of both toxicities and (2) *P. aquilinum* from serpentinite accumulates significantly more Ni and Cr than the same species from granite because it is adapted to soil containing increased concentrations of these metals.

## Materials and methods

### Study sites and collection of samples

**Fig. 1:**
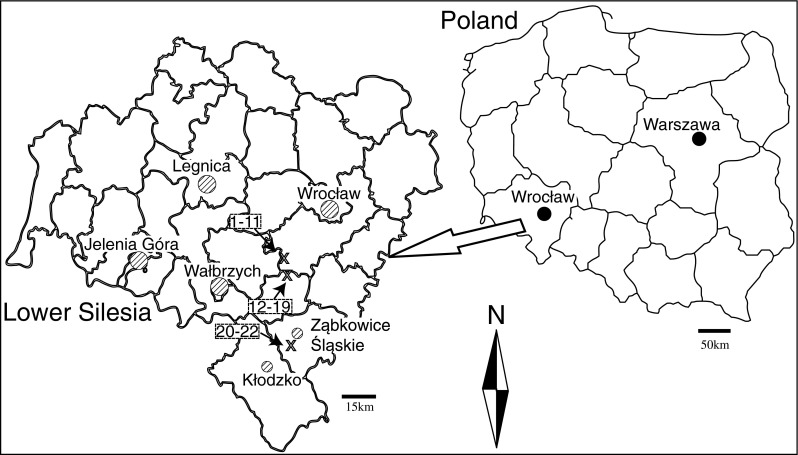
Map showing study areas and sampling locations (*x*)

In Lower Silesia (Fig. 1), a total of 22 sampling sites were selected, of which 11 on granites (sites 1–11) and 11 on serpentinites (sites 12–22). Soil profiles in those mountainous areas hardly accumulated organic material or developed a discernible A and B horizon because of erosion. Rock-forming minerals contain most of the nutrients required by plants for growth and development. Ground-weathered rock has been proposed as a source of slow release of the elements to be utilized by plants (Harley and Gilkes 2000). Therefore, in each site, pieces of parent rock material were collected. At each site, five samples of the rhizome and fronds were collected randomly within a 25 m × 25 m square. Each sample consisted of a mixture of three subsamples. As required by the rules set by the European Heavy Metal Survey (ICP Vegetation 2005), the collected ferns had not been exposed directly to canopy throughfall. Topsoil samples were also taken from each square, from a depth of 0 to 5 cm. Each sample consisted of a mixture of three subsamples. Plant remains and stones were removed from the soil. The total number of rock, soil, and plant (rhizome and frond) samples was 22 × 5 = 110. The selected areas were relatively pollution-free (WIOŚ 2009).

### Plant, parent rock, and soil analysis

Fresh soil samples were used for the determination of pH_H2O_ and pH_KCl_ potentiometrically (model: Hanna HI991300, Hanna Instruments Inc., Woonsocket (RI), USA) in a 1:2.5 soil-H_2_O and 1:2.5 soil-KCl ratios (Pansu and Gautheyrou 2006). Before analysis, rocks were crushed to a fine powder. Rhizomes were washed carefully for a few minutes and fronds for a few seconds in distilled water. Rock, soil, and plant samples were dried at 50 °C to constant weight. Soil samples were homogenized with a mortar and pestle after the coarse material was removed using a 2-mm sieve. Plant samples were homogenized to a fine powder in an IKA Labortechnik M20 laboratory mill. Dried soil and plant samples (300 mg of dry weight, in triplicate) were digested with 3 mL of nitric acid (ultra pure, 65 %) and 2 mL of perchloric acid (ultra pure, 70 %), and samples of rock powder (300 mg of dry weight, in triplicate) were digested in aqua regia in a CEM Mars 5 microwave oven. Samples were then diluted with deionized water to a total volume of 50 mL, and the soil and plant digests were analyzed for Fe, Mn, and Zn using FAAS and Cd, Co, Cr, Cu, Ni, and Pb using ETAAS with graphite furnace GF3000 (AVANTA PM Atomic Absorption Spectrophotometer from GBC Scientific Equipment). All elements were assayed against the atomic absorption standard solution from Sigma Chemical Co. and blanks containing the same matrix as the samples and were processed and analyzed as samples. Results of metal concentrations for the plants were calculated on a dry weight basis. The accuracy of the methods applied for the determination of the metal concentrations in plant and soil samples was checked against Certified Reference Materials: DC73348 LGC standards of bush branches and leaves and RTH 907 Dutch Anthropogenic Soil (Wageningen Evaluating Programs for Analytical Laboratories, WEPAL) (Table 1).

**Table 1:** Analysis of certified reference materials

Element	Certified	Found	Recovery	CV
	(mg kg^−1^)		(%)	
Bush branches and leaves DC73348 LGC				
Cd	0.14	0.13 ± 0.006	92.86	4.62
Co	0.39	0.38 ± 0.01	97.44	2.63
Cr	2.3	2.28 ± 0.06	99.13	2.63
Cu	5.2	5.05 ± 0.12	97.12	2.38
Fe	1,020	1,037 ± 23	101.67	2.22
Mn	58	58.33 ± 1.5	100.57	2.57
Ni	1.7	1.68 ± 0.06	98.82	3.57
Pb	7.1	6.78 ± 0.23	95.49	3.39
Zn	20.6	20.81 ± 0.32	101.02	1.54
Dutch Anthropogenic Soil RTH907				
Cd	2.18	2.22 ± 0.08	101.83	3.60
Cr	48.6	53.37 ± 1.22	109.81	2.29
Cu	121	119.63 ± 1.75	98.87	1.46
Pb	318	311.4 ± 7.53	97.92	2.42
Zn	714	716 ± 9.7	100.28	1.35
Co	9.09	8.98 ± 0.15	98.79	1.67
Fe	16,600	17,130 ± 630	103.19	3.68
Mn	506	527 ± 12	104.15	2.28
Ni	27.9	26.99 ± 1.03	96.74	3.82

### Experimental design

The toxicity of Cr and Ni ions on the fern (single metals as well as their combinations) was investigated. Cr and Ni as typical elements for serpentinites but also present in polluted environments were selected. From all the sampling sites analyzed, three on granite and three on serpentinite were chosen where the Cr and Ni concentration in the soil was equal to the average of all the investigated sites. Rhizomes of ferns were collected in March in a state of unfolded fronds (Zenkteler 1994). Rhizomes were cleaned from soil and washed in distilled water and then placed in threefold in pots filled with general potting mix soil according to Kachenko et al. (2007). Two groups of pots were established: one with “granite” rhizomes and second with “serpentinite” rhizomes. Each of them was divided into three subgroups which received Cr, Ni, and mixture of Cr and Ni, respectively. Metal salts were applied as 0 (control), 50, 100, and 250 mg kg^−1^ of dry weight of potting soil (for each metal) using K_2_Cr_2_O_7_ and NiCl_2_. The amounts were calculated by weight of elemental Cr and Ni (Kachenko et al. 2007). The hexavalent Cr(VI) species, considered the most toxic form, was selected (Shanker et al. 2005). Metal concentrations were chosen based on the average (~100 mg kg^−1^) concentration of Cr and Ni in serpentinite soils of the examined sites. One lower (50 mg kg^−1^) and one higher concentration (250 mg kg^−1^) were added in the test series to study the mutual metal interference at other than the average concentration. Pots were arranged in a completely randomized experimental design, and there were three replicates for each treatment. The plants were raised in a glasshouse for 20 weeks, watered daily with deionized water, and no fertilizer was applied during the experimental period (Kachenko et al. 2007). Ferns were harvested after 20 weeks and separated into rhizomes and fronds. Rhizomes were carefully cleaned, and rhizomes and fronds were washed with distilled water. Ni and Cr concentrations were established as described above. The total amount of samples was *N* = 216. The amount of replications was sufficient for proper statistical interpretation of the data.

### Statistical analysis

Differences among sampling sites with respect to metal concentrations in rocks, soil, rhizome, and fronds were evaluated by one-way ANOVA on log-transformed data to obtain the normal distribution of features according to Zar (1999). The normality of the analyzed features was checked by means of Shapiro-Wilk’s *W* test, and the homogeneity of variances was checked after transformation using the Brown-Forsythe test. Element concentrations in experimental rhizomes and fronds from granite and serpentinite sites were compared with *t* test (Zar 1999). All calculations were carried out using STATISTICA 10 software (StatSoft Inc. 2011).

## Results and discussion

The pH of the examined soils may be classified as acidic, significantly lower for granite than for serpentinite (Table 3). The pH is an important factor which influences the trace element bioavailability by affecting speciation and solubility and the properties of biological surfaces (Lithner et al. 1995). According to Blake and Goulding (2002) and Liu et al. (2005), bioavailability of Ni and Cr increases as soil pH decreases. So a lower pH may result in a higher solubility of both metals in the examined soils. The ranges of metal concentrations in parent rock, soil, and ferns are displayed in Tables 2, 3 and 4. The parent rock, soil, and fern samples differed significantly in terms of the concentrations of the elements assessed (ANOVA, *P* = 0.05). The concentrations (Table 2) of all the elements in granite were significantly lower than in serpentinite. This is in agreement with Galardi et al. (2007) that serpentine rocks are usually rich in Cr, Co, and Ni. The concentration of Co, Fe, Mn, and Zn in granite and serpentinite and of Cr and Ni in serpentinite was significantly higher than in overlaying soil (*t* test, *P* < 0.05). Higher metal concentrations in parent rocks than in overlaying soil were also observed by Samecka-Cymerman et al. (2011) in Lower Silesia. Rhizomes and fronds from ferns of serpentinite soils contained significantly higher concentrations of Cd, Co, Cr, and Ni and significantly lower concentrations of Mn than those from granite soils (Table 4). Thus, increased levels of metals in parent rocks and soils were reflected similarly in plants (Markert et al. 2003).

**Table 2:** Minimum, maximum, mean and standard deviation (SD), and *t* test of the concentration (mg kg^−1^) of metals in serpentinite and granite rock

	Serpentinite				Granite				*P* value
	Minimum	Maximum	Mean	SD	Minimum	Maximum	Mean	SD	
Cd	0.04	0.3	0.14	0.09	0.037	0.05	0.04	0.003	<0.01
Co	20	36	29	5.4	2.3	2.9	2.6	0.2	<0.001
Cr	171	412	232	89	2.2	6.5	4.1	1.4	<0.001
Cu	2.1	10	4.9	3	1.2	1.6	1.4	0.14	<0.01
Fe	28,305	60,548	50,953	12,034	7,176	11,857	9,467	1,714	<0.001
Mn	260	2,453	1,304	880	169	460	313	117	<0.01
Ni	696	1,273	920	200	1.5	3.4	2.4	0.6	<0.001
Pb	4.0	41	15	13	3.9	6.2	5.0	0.7	<0.05
Zn	26	94	56	24	31	42	35	3.2	<0.05

**Table 3:** Minimum, maximum, mean and standard deviation (SD), and *t* test of pH_H2O_, pH_KCl_, and the concentration (mg kg^−1^) of metals in soils of the serpentine and granite sites

	Serpentinite				Granite				*P* value
	Minimum	Maximum	Mean	SD	Minimum	Maximum	Mean	SD	
pH_H2O_	4.0	4.7	4.4	0.2	3.6	4.4	4.0	0.2	<0.001
pH_KCl_	3.3	4.3	3.8	0.4	3.0	3.9	3.3	0.3	<0.001
Cd	0.09	0.97	0.35	0.25	0.02	0.2	0.09	0.06	<0.001
Co	2.9	31	11	9.9	0.4	3.1	1.6	0.8	<0.001
Cr	44	193	99	47	3.9	14	8.1	3.1	<0.001
Cu	3.5	13	7.1	3.1	3.0	8.7	5.4	1.8	<0.05
Fe	5,971	14,222	9,203	2,643	3,254	8,604	6,207	1,451	<0.001
Mn	42	950	262	274	56	142	97	25	<0.01
Ni	31	191	101	47	2.1	7.9	4.5	1.6	<0.001
Pb	22	87	43	23	16	74	30	14	<0.05
Zn	18	75	35	22	9	38	18	8.6	<0.01

**Table 4:** Minimum, maximum, mean and standard deviation (SD), and *t* test of the concentration (mg kg^−1^) of metals in rhizome and fronds of *Pteridium aquilinum* from the serpentinite and granite sites

	Serpentinite				Granite				*P* value
	Minimum	Maximum	Mean	SD	Minimum	Maximum	Mean	SD	
Rhizome									
Cd	0.06	0.50	0.30	0.14	0.02	0.17	0.09	0.04	<0.001
Co	0.82	3.0	1.6	0.7	0.07	0.45	0.26	0.08	<0.001
Cr	0.70	11	6.1	3.4	1.4	2.5	1.9	0.32	<0.001
Cu	3.4	7.7	5.2	1.3	2.3	7.9	5.2	1.8	>0.05
Fe	140	282	205	45	82	271	154	52	<0.01
Mn	15	102	44	29	15	108	67	27	<0.01
Ni	11	29	19	5.7	1.9	8.1	3.7	1.4	<0.001
Pb	4.4	31	15	7.0	4.8	32	16	8.0	>0.05
Zn	20	42	30	5.9	9.2	33	23	8.0	<0.01
Fronds									
Cd	0.10	0.45	0.29	0.09	0.02	0.14	0.08	0.03	<0.001
Co	0.12	1.9	0.82	0.64	0.08	0.82	0.36	0.25	<0.01
Cr	1.2	3.7	2.6	0.83	1.2	2.6	1.7	0.45	<0.001
Cu	2.2	8.5	4.8	2.0	2.8	7.7	4.1	1.2	>0.05
Fe	60	149	105	19	66	130	102	16	>0.05
Mn	59	358	165	85	61	803	325	227	<0.01
Ni	4.6	19	9.4	5.1	1.6	11	3.9	2.4	<0.001
Pb	0.67	7.6	4.4	1.1	1.2	6.0	3.5	1.2	<0.05
Zn	19	45	28	7.4	12	39	24	7.5	>0.05

*P. aquilinum* sampled from serpentinite soils and those planted in the experimental pots contained significantly higher (*t* test, *P* < 0.05) concentrations of Cr and Ni in the rhizomes. According to Fargašova (2012), the accumulation of Cr was higher in roots than in upper plant parts. The transport of Ni to shoots was at least twice as high as that of Cr. Also, in this investigation, the concentration of Ni in fronds was at least twice as high as that of Cr. According to Oze et al. (2008), serpentinite vegetation suppresses Cr and Ni uptake into its aboveground biomass, and both elements are preferentially immobilized or sequestered in roots rather than leaves. This is probably a major mechanism for serpentinite vegetation to tolerate the elevated levels of metals in soils (Oze et al. 2008). Kachenko et al. (2007) also report that metal translocation was limited because of absorption and retention in roots, suggesting an exclusion mechanism as part of the ferns’ tolerance to the metals supplied. Additionally, Cr compared to Ni is usually strongly bound in serpentinite soils, and the concentration of this element in roots was well below those in soils. However, nickel concentrations in plants usually reflect those in soil (Kabata-Pendias 2001). Plant tissue bioaccumulation factor (plant tissue element concentration) / (plant soil element concentration) of Ni for *P. aquilinum* from serpentinite and granite sites investigated by Samecka-Cymerman et al. (2009) exceeded 1, indicating that *P. aquilinum* is not an Ni excluder. There was also a significant positive Pearson correlation between the concentration of Ni in soil and Ni in *P. aquilinum*.

**Fig. 2:**
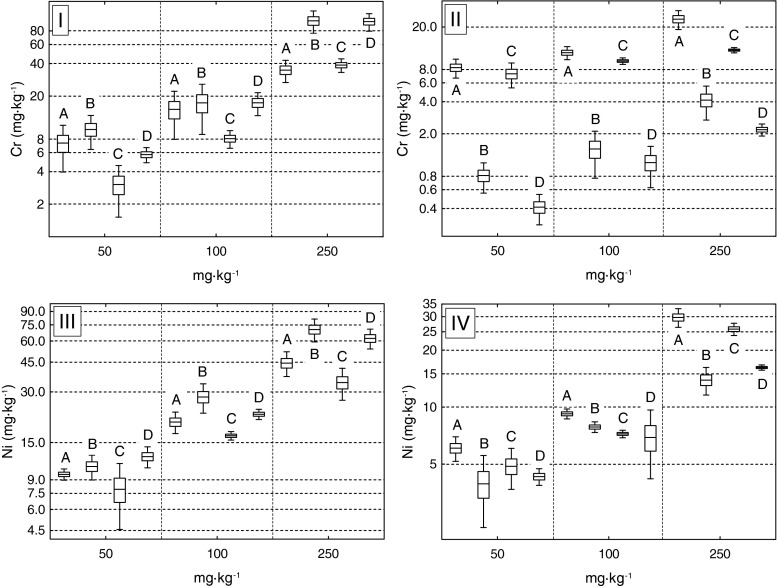
Cr or Ni concentrations in rhizomes and in fronds of *P. aquilinum* from granite and serpentinite cultivated in experimental soils with concentrations of 50, 100, and 250 mg kg^−1^ of Cr and Ni applied separately or combined. *I* Cr in rhizome, *II* Cr in fronds, *III* Ni in rhizome, *IV* Ni in fronds; *A* serpentinite combined, *B* serpentinite separate, *C* granite combined, *D* granite separate, – mean,  standard error,  confidence interval

There was no difference in the concentration of Ni and Cr (applied both separately or together; all treatments) between the serpentinite and granite *P. aquilinum* rhizome and fronds (*t* test, *P* < 0.05). The exception was Cr concentration in fronds (Fig. 2) which was significantly higher in serpentinite than in granite ferns. This is in agreement with Żołnierz (2007) that serpentinite species frequently accumulate vast quantities of Cr. This lack of difference in Cr and Ni concentration may probably indicate that the phenotypic plasticity of this species is very important in the adaptation to extreme environments and probably increases the survival of *P. aquilinum* in contaminated sites (Eränen 2008; Dunn and Rothwell 2012).

There was a significant increase in the concentration of Cr and Ni in the experimental ferns with higher applications of both elements; however, a sharp increase in metal accumulation was observed at concentrations higher than 100 mg kg^−1^ (Fig. 2). According to Kachenko et al. (2007), such a phenomenon suggests breakdown in tolerance mechanisms and unrestricted metal transport. The results of the experiment were most spectacular for the 250 mg kg^−1^ level. In this concentration, rhizomes (both granite and serpentinite) contained significantly higher Cr and Ni concentrations when both metals were supplied separately (post hoc least significant difference (LSD), *P* < 0.05) (Fig. 2). However, fronds (both granite and serpentinite) contained significantly higher concentrations of Cr and Ni when both metals were supplied together (post hoc LSD, *P* < 0.05). According to Shah et al. (2010), Ni and Cr showed good correlation in both soils and plants in serpentinite areas. Baker et al. (1999) and Samecka-Cymerman et al. (1997) also suggest that the combined toxicity of both metals might be higher than the sum of the individual toxicities of each separate element. These results are in agreement with Shanker et al. (2005) that the toxicity of some elements changes in the presence of other metals and results in different effects comparing rhizomes with stems and leaves because of changing ratios. A Cr/Ni different effect in fronds and rhizomes is therefore possible for metal uptake in *P. aquilinum*. Thus, Cr and Ni uptake in the fern appears to be controlled by their mutual concentrations (Ondo et al. 2012)*.* According to Tomasik et al. (1995), the same Cr-Ni metal ions are listed as mitigating transfer from the soil to rhizome level and favoring transfer from the rhizome to fronds. The explanation of differences in the joint accumulation of Cr and Ni on the rhizome or frond level may probably be the competitive binding of Cr and Ni to the rhizome cell wall (Glick 2003). The interaction between ferns and rhizosphere microorganisms or mycorrhizal symbiosis which may influence the tolerance of *P. aquilinum* for metals should not be neglected (Khan 2001). There is a possibility that both elements may be accumulated in the extrametrical hyphae or excluded by the symbiont (Göhre and Paszkowski 2006). However, to explain these differences in metal accumulation, further investigation is needed. The differences in the Cr accumulation behavior in the presence of Ni and other metals in plants need further investigation.

## Conclusions

*P. aquilinum* sampled from serpentinite soils and those planted in the experimental pots contained significantly higher concentrations of both elements in the rhizome than in fronds. Grown at a concentration of 250 mg kg^−1^ of Cr or Ni in soil, fronds (both granite and serpentinite) contained significantly higher Cr and Ni concentrations when both metals were supplied together. At a concentration of 250 mg kg^−1^ of Cr or Ni, rhizomes (both granite and serpentinite) contained significantly higher Cr and Ni concentrations when both metals were supplied separately. To explain these differences, further investigation is needed. In experimental ferns, there was no difference in the concentration of Ni and Cr (applied both separately or together; all treatments) between the serpentinite and granite *P. aquilinum* rhizome and fronds. The exception was Cr concentration in fronds which was significantly higher in serpentinite than in granite ferns. This lack of difference may probably indicate that the phenotypic plasticity of this species is very important in the adaptation to extreme environments and probably increases the survival of this fern in contaminated sites.

The results of this investigation may be applied in the bioindication of Ni and Cr in anthropogenic polluted environments. It contributes to the use of *P. aquilinum* in bioindication of the combined presence of Cr and Ni, taking into account their changing effects at different ratios in the environment. Further investigation might supply assertion which biogeochemical condition will promote efficient phytoremediation by this species.

## References

[CR1] Baker AJM, McGrath SP, Reeves RD, Smith JAC, Terry N, Bañuelos GS (1999). Metal hyperaccumulator plants: a review of the ecology and physiology of a biological resource for phytoremediation of metalpolluted soils. Phytoremediation of contaminated soil and water.

[CR2] Blake L, Goulding KWT (2002). Effects of atmospheric deposition, soil pH and acidification on heavy metal contents in soils and vegetation of semi-natural ecosystems at Rothamsted Experimental Station, UK. Plant Soil.

[CR3] Chang JS, Yoon IH, Kim KW (2009). Heavy metal and arsenic accumulating fern species as potential ecological indicators in As-contaminated abandoned mines. Ecol Indic.

[CR4] Dunn MT, Rothwell GW (2012). Phenotypic plasticity of the hydrasperman seed fern *Tetrastichia bupatides* Gordon (Lyginopteridaceae). Int J Plant Sci.

[CR5] Eränen JK (2008). Rapid evolution towards heavy metal resistance by mountain birch around two subarctic copper–nickel smelters. J Evol Biol.

[CR6] Fargašova A (2012). Plants as models for chromium and nickel risk assessment. Ecotoxicology.

[CR7] Fernándèz JA, Rey A, Carballeira A (2000). Differences in the responses of native and transplanted mosses to atmospheric pollution: a possible role of selenium. Environ Pollut.

[CR8] Galardi F, Mengoni A, Pucci S, Barletti L, Massi L, Barzanti R, Gabbrielli R, Gonnelli C (2007). Intra-specific differences in mineral element composition in the Ni-hyperaccumulator *Alyssum bertolonii*: a survey of populations in nature. Environ Exp Bot.

[CR9] Glick BR (2003). Phytoremediation: synergistic use of plants and bacteria to clean up the environment. Biotechnol Adv.

[CR10] Göhre V, Paszkowski U (2006). Contribution of the arbuscular mycorrhizal symbiosis to heavy metal phytoremediation. Planta.

[CR11] Haiyan W (2003). Effect of Cd, Zn, and Pb compound pollution on celery in a ferric acrisol. Soil Sediment Contam.

[CR12] Harley AD, Gilkes RJ (2000). Factors influencing the release of plant nutrient elements from silicate rock powders: a geochemical overview. Nutr Cycl Agroecosyst.

[CR13] ICP Vegetation: Heavy metals in European mosses: 2005/2006 survey (2005). Monitoring manual. UNECE ICP Vegetation Coordination Centre.

[CR14] Kabata-Pendias A (2001). Trace elements in soils and plants.

[CR15] Kachenko AG, Singh B, Bhatia NP (2007). Heavy metal tolerance in common fern species. Aust J Bot.

[CR16] Khan AG (2001). Relationships between chromium biomagnification ratio, accumulation factor, and mycorrhizae in plants growing on tannery effluent-polluted soil. Environ Int.

[CR17] Leung HM, Ye ZH, Wong MH (2007). Survival strategies of plants associated with arbuscular mycorrhizal fungi on toxic mine tailings. Chemosphere.

[CR18] Lithner G, Holm K, Borg H (1995). Bioconcentration factors for metals in humic waters at different pH in the Ronnskar area (N. Sweden). Water Air Soil Pollut.

[CR19] Liu H, Probst A, Liao B (2005). Metal contamination of soils and crops affected by the Chenzhou lead/zinc mine spill (Hunan, China). Sci Total Environ.

[CR20] Markert BA, Breure AM, Zechmeister HG, Markert BA, Breure AM, Zechmeister HG (2003). Definitions, strategies and principles for bioindication/biomonitoring of the environment. Bioindicators and biomonitors.

[CR21] Marrs RH, Watt AS (2006). Biological flora of the British Isles: *Pteridium aquilinum* (L.) Kuhn. J Ecol.

[CR22] Niazi NK, Singh B, Van Zwieten L, Kachenko AG (2012). Phytoremediation of an arsenic-contaminated site using *Pteris vittata* L. and *Pityrogramma calomelanos* var. *austroamericana*: a long-term study. Environ Sci Pollut Res.

[CR23] Ondo JA, Prudent P, Menye Biyogo R, Domeizel M, Vassalo L, Eba F (2012). Effects of Cu and Zn supplementation on metal uptake by *Hibiscus sabdariffa*. Res J Chem Sci.

[CR24] Oze C, Skinner C, Schroth AW, Coleman RG (2008). Growing up green on serpentine soils: biogeochemistry of serpentine vegetation in the Central Coast Range of California. Appl Geochem.

[CR25] Pansu M, Gautheyrou J (2006). Handbook of soil analysis: mineralogical, organic and inorganic methods.

[CR26] Pivetz B (2001) Phytoremediation of contaminated soil and ground water at hazardous waste sites. Ground Water Issue, U.S. Environmental Protection Agency, Office of Research and Development and Office of Solid Waste and Emergency Response EPA/540/S-01/500: 1-36

[CR27] Roy BK, Gunjan PR (2010). Heavy metal accumulation and changes in metabolic parameters in *Cajanas cajan* grown in mine spoil. J Environ Biol.

[CR28] Samecka-Cymerman A, Marczonek A, Kempers AJ (1997). Bioindication of heavy metals in soil by liverworts. Arch Environ Contam Toxicol.

[CR29] Samecka-Cymerman A, Garbiec K, Kolon K, Kempers AJ (2009). Factor analysis of the elemental composition of *Pteridium aquilinum* from serpentine and granite soils as a tool in the classification of relations between this composition and the type of parent rock in the Ślęża Massif in Lower Silesia, Poland. Environ Geol.

[CR30] Samecka-Cymerman A, Kolon K, Stankiewicz A, Kaszewska J, Mróz L, Kempers AJ (2011). Rhizomes and fronds of *Athyrium filix-femina* as possible bioindicators of chemical elements from soils over different parent materials in southwest Poland. Ecol Indic.

[CR31] Samecka-Cymerman A, Kolon K, Stankiewicz A, Mróz L, Kempers AJ (2012). Bioindicative comparison of the fern *Athyrium distentifolium* for trace pollution in the Sudety and Tatra mountains of Poland. Environ Monit Assess.

[CR32] Shah MT, Begum S, Khan S (2010). Pedo and biogeochemical studies of mafic and ultramfic rocks in the Mingora and Kabal areas, Swat, Pakistan. Environ Earth Sci.

[CR33] Shanker AK, Cervantes C, Loza-Taverac H, Avudainayagam S (2005). Chromium toxicity in plants. Environ Int.

[CR34] StatSoft Inc (2011) STATISTICA (data analysis software system), version 10. <www.statsoft.com>

[CR35] Tomasik P, Magadza CM, Mhizha S, Chirume A, Zaranyika MF, Muchiriri S (1995). Metal-metal interactions in biological systems. Part IV. Freshwater snail *Bulinus globosus*. Water Air Soil Pollut.

[CR36] WIOŚ Wojewódzki Inspektorat Ochrony Środowiska (2009) Stan środowiska województwa dolnośląskiego w 2008 roku [State of environment in Lower Silesia in 2008]

[CR37] Zar H (1999) Biostatistical analysis. In: Ryo T (ed.) fourth ed. Prentice-Hall Inc., Upper Saddle River

[CR38] Zenkteler E (1994). Paprocie [Ferns].

[CR39] Zhang S, Li T, Huang H, Zou T, Zhang X, Yu H, Zheng Z, Wang Y (2012). Cd accumulation and phytostabilization potential of dominant plants surrounding mining tailings. Environ Sci Pollut Res.

[CR40] Żołnierz L (2007). Grasslands on serpentines in lower Silesia (SW Poland)—some aspects of their ecology. Zesz Nauk Uniw Przyrodniczego we Wrocławiu.

